
JOURNAL INFORMATION
==============================
NLM Title Abbreviation: ScientificWorldJournal
Journal ID: ScientificWorldJournal
NLM Journal ID: 101131163
Journal ID: TSWJ

The Scientific World Journal

ISSN: 2356-6140
EISSN: 1537-744X
Publisher: Wiley

ARTICLE INFORMATION
==============================
PMCID: PMC6009282
PMID: 29973775
DOI: 10.1100/tsw.2002.1
Article ID: 149537
Article version: 1
Subjects: Short Report

Microbial Genome Sequencing: Prospects for Development of Novel Vaccines and Anti-Microbial Compounds

Fraser Claire M. *
The Institute for Genomic Research, 9712 Medical Center Drive, Rockville, MD 20850, USA
*Claire M. Fraser: cmfraser@tigr.org
Publication date: 2002
Electronic publication date: 2002 Jan 16
Volume: 2
First page: 1
Last page: 2
Copyright: Copyright © 2002 Claire M. Fraser.
Copyright year: 2002
License: This is an open access article distributed under the Creative Commons Attribution License, which permits unrestricted use, distribution, and reproduction in any medium, provided the original work is properly cited.
License URL: https://creativecommons.org/licenses/by/3.0/

==============================
Advances in Gene Technology: The Genome and Beyond –
Structural Biology for Medicine (Proceedings of the 2002 Miami
*Nature Biotechnology* Winter Symposium)
*TheScientificWorld* 2002, 2(S2), 1–2
ISSN 1532-2246; DOI 10.1100/tsw.2002.1

### MICROBIAL GENOME SEQUENCING: PROSPECTS FOR DEVELOPMENT OF NOVEL VACCINES AND ANTI-MICROBIAL COMPOUNDS

Claire M. Fraser and the Microbial Genome Sequencing and Annotation Groups
The Institute for Genomic Research, 9712 Medical Center Drive, Rockville, MD 20850
cmfraser@tigr.org

Since the first genome sequence for a free-living organism, *Haemophilus influenzae*, was published in 1995[1], more than 50 microbial sequencing projects have been completed. Work is underway on more than 100 microbial genome projects from a diverse group of pathogens, archaea, and species of evolutionary importance (see http://www.tigr.org for a complete list). In the next 2–3 years, international efforts in microbial genome sequencing will generate more than 250 Mbp of DNA sequence containing ~300,000 predicted genes, several times the number of genes expected from completion of the human genome project. Genome sequencing still remains the most robust method for assessing the overall gene complement of any organism and as costs for DNA sequencing have dramatically decreased, the possibility of using this approach to study new species and microbial populations has become more realistic. It is important to keep in mind that all of the work done to date in microbial genomics has focused on species that can be cultured in the laboratory or grown in animal cells. However, uncultured species should be a priority for future genome studies and the technology now exists to allow us to think about microbial community genome projects.

Microbial genome sequence data has begun to reveal an enormous biochemical and metabolic complexity in the microbial world. Almost 50% of all predicted coding sequences identified to date are of unknown biological function and approximately 10% of all predicted coding sequences in each species studied with genome analysis to date are unique, having no significant sequence similarity to any other available protein sequence. Taken together, these data indicate that there is a substantial amount of microbial biology still be elucidated and suggest that the idea of a “model organism” in the microbial world may not be valid, given the vast differences that are observed, even between related species. A significant proportion of larger microbial genomes represent paralogous genes, that is, genes related by duplication rather than by vertical inheritance, and these represent some of the most interesting genes in each species, often with specialized functions. Comparative genomics has suggested that lateral gene transfer both between and among bacterial and archaeal species has been more extensive than was previously described and this process has also played an important role in generating species diversity. It is now clear that species evolution cannot be predicted from the study of single gene phylogenies.

Genomics approaches show great promise in our ability to understand and treat infectious disease. The recent completion of the *Neisseria menigitidis* genome sequence[2] has demonstrated that genome information can be exploited in the rapid identification of novel vaccine candidates[3].

This search is being aided by technologies such as DNA arrays that allow for a comprehensive survey of large numbers of clinical isolates to identify differences among closely related organisms as well as insights into the most important genes in pathogens and hosts for initiating and maintaining infection.

### REFERENCES

1. Fleischmann, R.D., Adams, M.D., White, O., Clayton, R.A., Kirkness, E.F., et al. (1995) Science 269, 496–512.
2. Tettelin, H., Saunders, N.J., Heidelberg, J., Jeffries, A.C., Nelson, K.E., et al. (2000) Science 287, 1809–1815.
3. Pizza, M., Scarlato, V., Masignani, V., Giuliani, M.M., Arico, B., et al. (2000) Science 287, 1816–1820.
